# A Comparison of the Number of Men Who Have Sex with Men among Rural-To-Urban Migrants with Non-Migrant Rural and Urban Residents in Wuhan, China: A GIS/GPS-Assisted Random Sample Survey Study

**DOI:** 10.1371/journal.pone.0134712

**Published:** 2015-08-04

**Authors:** Xinguang Chen, Bin Yu, Dunjin Zhou, Wang Zhou, Jie Gong, Shiyue Li, Bonita Stanton

**Affiliations:** 1 Department of Epidemiology, University of Florida, Gainesville, Florida, United States of America; 2 Wuhan Centers for Disease Prevention and Control, Wuhan, China; 3 School of Public Health, Wuhan University, Wuhan, China; 4 Pediatric Prevention Research Center, Wayne State University, Detroit, Michigan, United States of America; UCSF, UNITED STATES

## Abstract

**Background:**

Mobile populations and men who have sex with men (MSM) play an increasing role in the current HIV epidemic in China and across the globe. While considerable research has addressed both of these at-risk populations, more effective HIV control requires accurate data on the number of MSM at the population level, particularly MSM among migrant populations.

**Methods:**

Survey data from a random sample of male rural-to-urban migrants (aged 18-45, n=572) in Wuhan, China were analyzed and compared with those of randomly selected non-migrant urban (n=566) and rural counterparts (580). The GIS/GPS technologies were used for sampling and the survey estimation method was used for data analysis.

**Results:**

HIV-related risk behaviors among rural-to-urban migrants were similar to those among the two comparison groups. The estimated proportion of MSM among migrants [95% CI] was 5.8% [4.7, 6.8], higher than 2.8% [1.2, 4.5] for rural residents and 1.0% [0.0, 2.4] for urban residents, respectively. Among these migrants, the MSM were more likely than non-MSM to be older in age, married, and migrated to more cities. They were also more likely to co-habit with others in rental properties located in new town and neighborhoods with fewer old acquaintances and more entertainment establishments. In addition, they were more likely to engage in commercial sex and less likely to consistently use condoms.

**Conclusion:**

Findings of this study indicate that compared to rural and urban populations, the migrant population in Wuhan consists of a higher proportion of MSM who also exhibit higher levels of HIV-related risk behaviors. More effective interventions should target this population with a focus on neighborhood factors, social capital and collective efficacy for risk reduction.

## 1 Introduction

### Men who have sex with men and the HIV epidemic

Men who have sex with men (MSM) have prominent role in today’s HIV epidemic in many countries across the world, including China [1–3]. The documented prevalence rates of HIV/AIDS are high among MSM, typically ranging from 5–15%, but with some samples having substantially higher rates [4]. For instance, a HIV prevalence rate of 32.9% was documented among a sample of 641 MSM in Zambia [5]. In the United States, MSM accounted for 57% of new infections in 2006 [6], and increased to 64% in 2011 [7]. Sexual transmission also plays a critical role in the HIV epidemic in China [8]. Data from the national sentinel stations indicate that the percentage of sexually transmitted HIV infections increased from 11.6% in 2005 to 76.3% in 2011, and the proportion of HIV infections among MSM increased 45-fold in six years from 0.3% in 2005 to 13.7% in 2011 [9], and one-third of new HIV infections are from MSM [9]. Studies conducted in Wuhan, China indicate that the HIV prevalence rate among MSM varied from 4.4% to 6.6% [10–13].

### Extra high risk sex of HIV infection among MSM

Compared to the general population, HIV-related risk behaviors among MSM are more prevalent, including unprotected sex, illicit drug use, alcohol consumption, and tobacco smoking [14, 15]. Many HIV risk behaviors among MSM observed in other countries are also reported in China. These behaviors include anal sex, multi-partners sex [16], commercial sex [17], sex without using a condom [18, 19], alcohol consumption, drug use and tobacco smoking [14]. For example, a survey study among MSM (n = 405) in Harbin, northern China indicates that 71.5% of the sample do not practice consistent condom use and 22.6% engage in commercial sex [17]. One survey study (n = 464) conducted in Wuhan revealed high rates of engagement in commercial sex (40.09%) and inconsistent condom use (62.07%) among MSM [12].

Anal sexual intercourse without using a condom further increases the risk of HIV infection because the anal canal has histological structures that are more vulnerable than the vaginal canal to viral penetration [20]. The documented successful HIV infection rate per anal sex act is 1.4%, 95% CI [0.2–2.5], which is 18 times higher than that through vaginal sex (0.08%, 95% CI [0.06–0.11]) [21, 22]. If more rural-to-urban migrants have engaged in MSM than non-migrant rural and urban residents, we have evidence to conclude that rural migrants are a high risk population to control the HIV epidemic in China.

### MSM among rural-to-urban migrants in China

Since the 1980s when China started experiencing rapid economic growth, a large number of farmers have migrated from rural areas to urban areas to earn wages. This population, currently totaling approximately 260 million [23] presents a great public health challenge. Some studies report a higher prevalence of HIV risk behaviors among rural migrants than non-migrant rural and urban residents, including sexual risk behaviors [24–27], alcohol use and abuse [24, 25], and tobacco smoking [28]; other studies indicate that rural migrants may be less likely than their non-migrant counterparts in rural and urban areas to engage in HIV risk behaviors [29, 30].

The majority of sex workers in China are rural-to-urban migrants, including both female and male sex workers [31]. However, little is known specifically about MSM among the rural migrant population who frequently shuttle between urban residential areas where they live and work and rural homes from where they originate. When leaving their rural homes, migrants lose their rural-rooted social capital [32, 33], exposing them to a large array of environmental and behavioral factors many of which carry great risk. Relative to non-migrant urban residents, male rural migrants may be more likely to engage in same-gender sex [34]. These migrants are typically married but migrate alone to cities, reducing their connections with families and societies in their rural homes [35]. While settling down in a city, they typically live in poorer neighborhoods with other migrants of the same gender from the same villages as roommates, separating them from the mainstream urban context [36]. MSM may have more opportunities to access to entertainment settlements in urban areas, increasing the likelihood for them to engage in HIV risk behaviors [37, 38].

### Challenges to estimate the number of MSM

Despite the growing significance of MSM in the HIV epidemic, there is a lack of knowledge regarding the number of MSM in China, particularly MSM among the rural-to-urban migrant population. Effective HIV prevention planning and decision-making would be incomplete without such data. As of the time when this study was completed, there are still no national estimates of the number of MSM in China. A few studies have suggested a higher proportion of MSM among rural migrants [34, 39]. For example, one study with a MSM sample (n = 500) in Beijing indicate that 81.8% of MSM were rural-to-urban migrants [39]. A couple of population-based studies suggest that MSM may account for 2–4% of the Chinese adult male population [40, 41]. However, none of these estimates was based on random samples.

Various methods are available to sample mobile and hidden populations, including migrants and MSM, such as venue-day-time sampling [42], respondent-driving sampling [43, 44], and capture-recapture [45, 46]. However, the validity of these methods in ensuring random samples has to be evaluated. Recent rapid advancement in geographic information systems (GIS) and global positioning systems (GPS) technologies provides new opportunities to draw random samples to quantify the number of MSM among rural-to-urban migrants. One reported study has attempted a GIS/GPS-assisted method to draw a random sample of rural migrants in Beijing, China [47]. Although the sampling procedure was terminated due to the large number of eligible participants in a few geounits, this research provides valuable experience for devising better GIS/GPS-assisted random sampling methods for mobile populations, such as the population of rural-to-urban migrants.

### Purpose of this project

The purpose of this study is three folds: The first and most important aim is to estimate the number of MSM among a random sample of rural-to-urban migrants in Wuhan, a typical provincial capital city in China with an average level of development; the second aim is to characterize HIV risk behaviors of migrant MSM by contrasting MSM with non-MSM; and the last aim is to compare migrants with non-migrant rural and urban residents with regard to the number of MSM and their risk behaviors. The ultimate goal is to provide data supporting decision making and prevention intervention measures targeting migrant population, particularly migrant MSM for more effective HIV control.

## 2 Materials and Methods

### 2.1 Ethics statement

The Institutional Review Boards’ approval of the study was obtained from Wuhan Center for Disease Prevention and Control, Wuhan, China, the Wayne State University, Detroit, and the University of Florida, Gainesville, USA. Written consent was obtained from all the participants before the survey.

### 2.2 Target population and study participants

The target population was rural-to-urban migrants aged 18–45 who were legal to work and at high risk of HIV infection. For comparison purposes, non-migrant rural and urban residents in the same age range were included. To enhance effective comparisons, we targeted the non-migrant urban residents who lived in the same or nearby areas where the selected rural migrants live, and the non-migrant rural residents who reside in places from which most rural migrants originate.

**Fig 1:**
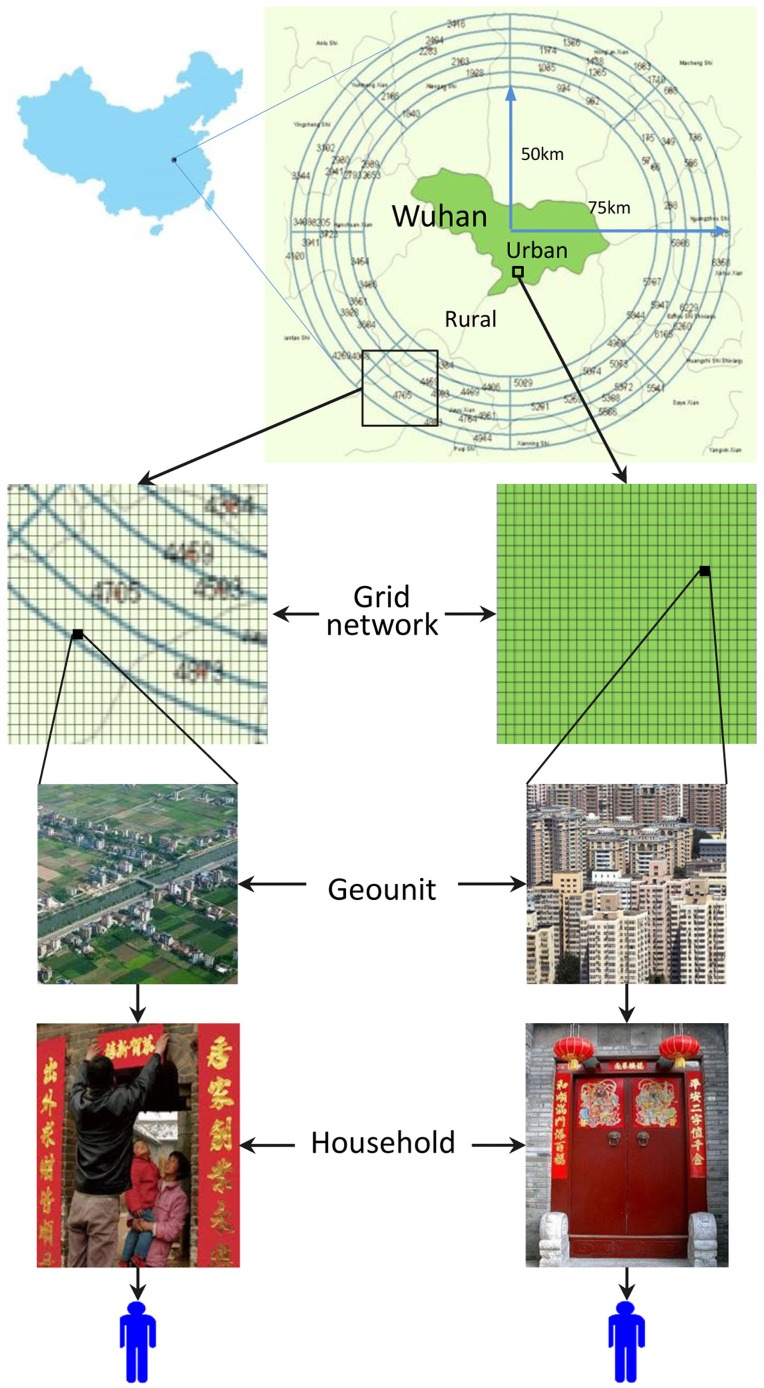
Scheme of GISGPS-Assisted Sampling.

Eligible rural migrants were defined as those who had a rural *Hukou* (legal rural residence), migrated to the city to earn money, and had stayed in the current city for at least one month. Likewise, non-migrant urban residents were defined as having urban *Hukou* (legal urban residence), had lived in the current city for at least five years. Non-migrant rural residents were defined as legal rural residents who staying in their rural homes and had not moved to urban areas to earn money in the past 12 months. Findings from our pilot studies indicated that farmers who had not migrated to city to make money in the last 12 months were unlikely to have migrated in the previous years. All participants were sampled in Wuhan (Fig 1), the capital city of Hubei Province with a total population of 10 million and GDP per capital of $12,708 in 2012 and large number of rural-to-urban migrants [48].

### 2.3 GIS/GPS-assisted random sampling

The participants were selected using the novel GIS/GPS-assisted sampling method we devised based on the spatial random sampling method [47]. Fig 1 summarizes the sampling procedure. The sampling was completed by the trained research staff from Wuhan CDC in the following four steps: (a) The residential areas of Wuhan where the target population reside were divided with a grid network into small and mutually exclusive cells named “geounits” using the GIS techniques on computer and these geounits were thus used to construct the primary sampling frame (PSF); (b) geounits were thus randomly selected from the PSF stratified by districts of Wuhan, and the information regarding these geounits were uploaded to a GPS receiver, including maps of the geounits and their surrounding areas, transportation routes, and health care and administrative agencies at the local level. (c) a small team consisting of a project coordinator, a GIS/GPS expert and a senior research staff with field data collection experience went to the field to physically locate the sampled geounits one by one with the assistance of the loaded GPS receiver, to obtain support from local health workers and community leaders and to plan for data collection; and (d) on a pre-scheduled date, a team of trained data collectors consisting of 5–6 senior research staff from Wuhan CDC and 8–10 graduate students was dispatched to the site to enumerate the households located within the sampled geounit, create the secondary sampling frame (SSF), and randomly sample participants from the SSF. To enhance independence, one participant per household was selected. For households with more than one eligible participant, only one was selected randomly.

To sample rural-to-urban migrants and non-migrant urban residents, the sample size, the number of participants per geounit and the total number of geounits were determined in the following three steps: (1) The sample size was determined following the stratified multilevel randomized design [49, 50]. Using the software nQuery Advisor version 5.0 (Statistical Solutions Ltd, Boston, MA, USA) an estimate of N = 600 was adequate to determine the prevalence of MSM with ±5%. (2) The number of participants per geounit was determined using the optimal allocation strategy[49, 50],strategy considering intraclass correlation and the ratio of the traveling cost over the total cost (traveling, household enumeration and interview). With the intraclass correlation set at .02-.03 and the cost ratio set at .3, the estimated number of participants per geounit was 10. (3) The number of geounits to be sampled was determined by dividing the total sample (N = 600) with the number of participants per geounit (n = 10) or 60 geounits.

To implement the sampling plan, the urban areas of Wuhan were first divided into mutually exclusive geounits of 100 meters by 100 meters as the PSF. The geounit size was determined through repeated pilot tests to ensure an appropriate number of households per unit for sampling and to be cost-effective in term of participant recruitment and data collection. The 60 urban geounits were thus randomly selected from the PSF and allocated to the seven urban districts of Wuhan using the *optimal design method* such that relatively more geounits were allocated to districts with higher population density [49]. The same number of migrants and non-migrant urban residents were randomly sampled from different households within the same geounits.

The same GIS/GPS-assisted procedure was modified for sampling rural residents. The target residential areas of rural residents were defined as a band surrounding the urban core of Wuhan, with a band width of 25 kilometers and inner radius of 50 kilometers, representing the places from which most rural migrants in Wuhan originated. The targeted band region was divided into mutually exclusive geounits with the size 1 kilometer by 1 kilometer to create the PSF. This geounit size was determined based on preliminary tests in several typical rural regions (e.g., plains, hills, mountains, and lakes) to ensure 90% likelihood of coverage of at least one rural village per geounit. Given the increased traveling cost, we sampled 40 geounits from the PSF with 15 participants being distributed to each geounit to produce approximately 600 male participants. The 40 geounits were randomly distributed into 40 strata with one unit per strata. The strata were created by dividing the whole band region with four co-centric circles 5-kilometer apart and four evenly distributed straight lines through the origin.

The commercial software ArcGIS, version 10.0 (ESRI, Inc, Redlands, CA) was used to sample geounits. The GPS receiver (Garmin Oregon 450, Garmin, Ltd) was used to assist in locating the sampled geounits and to assess the actual area size of a geounit from which households were sampled. To ensure adequacy of sampling, 20% extra geounits were added.

### 2.4 Procedures and data collection

Field data collection was carried out by the Wuhan Center for Disease Prevention and Control (CDC) from March 2011 to December 2013. Participant sampling and data collection of all sampled geounits were completed one by one through an organized strategy. First, a pre-survey team consisting of one leader (typically the director or the deputy director of Wuhan CDC), one GIS/GPS expert, and one senior staff was dispatched to a sampled geounit. The goals of the trip were to make contact with grass-root level administrative agencies and/or a health center closest to the selected geounit to obtain their support, to work together with them to physically assess the feasibility of sampled geounit, and to plan for data collection.

On the pre-determined survey date, a team of data collectors (typically 4 to 5 senior research staffs, plus 8 to 10 graduate students) was dispatched to the site for subject recruitment and data collection. Survey data were collected with the Migrant Health and Behavior Questionnaire [29], delivered using Audio Computer-Assisted Self Interviewing (ASACI). The survey was conducted in a designated room located in the participants’ home or a local health center. A brief ACASI training was provided before completing the survey on computer. Data collectors were available for assistance while participants were completing the survey. At the completion, participants received material rewards with a value of 5–6 UD dollars.

Among the 4215 eligible participants approached, 261 (6%) refused to participate and 3954 completed the survey, of whom 1939 (49%) were male. Toward the end of the survey, all participants were asked to indicate the level of reliability of the answers they provided to all questions they completed with 1 = 100% reliable; 2 = 80% reliable; 3 = 50% reliable; 4 = 20% reliable; and 5 = totally unreliable. Among the 1939 males, 221 (11%) responded 3 or higher were excluded, yielding a final sample of 1718.

### 2.5 Measurements

#### Demographic, migration, living conditions and neighborhood environment

(a) Four demographic variables were assessed for all participants, including age (in years), marital status (married, unmarried), education (primary, middle school, high school, college or more), and monthly income (RMB, <1000, 1000–2000, 2000–4000, >4000). (b) Four variables for assessing living conditions were: residential locations (old town, new town, rural-urban joint zone, and suburban), housing ownership (owned, rented and others), living arrangement (alone or co-habit), and intention to move in the future (likely, unsure, and unlikely). (c) Five variables for assessing neighborhood conditions were: perceived safety (safe, unsure, unsafe), proportion of urban residents (<half, about a half, >half), prior acquaintances (yes/no), existence of (none, some, a lot) and accessibility (convenient, unsure, inconvenient) to entertainment venues in neighborhoods. (d) Four variables only for migrants were: the number of cities ever migrated to (1, 2–3, and ≥4 cities), years of migration, number of home visits per year (0, 1–2 and ≥3) and if sent money home (yes/no) in the past year.

#### MSM, commercial sex and condom use

Data used to assess MSM status, engagement in commercial sex and condom use were collected and further analyzed. During the survey, participants were asked “Have you ever engaged in sexual intercourse with any of the following persons?” A list of six categories of persons were included as *multiple choices*: “sex workers”, “drug users”, “blood donors”, “persons infected with HIV”, “persons infected with STD” and “same gender persons”. Participants were coded as MSM if they reported ever having had sex with a same gender person [51], and as having engaged in commercial sex if they reported having had sex with sex workers. Other risk partners were not analyzed because of the limited number of positive answers.

Participants were further asked: “How often do you use a condom when you have sex with any of these persons?” A four-level frequency scale (1 = “Never”, 2 = “Occasional”, 3 = “Often”, and 4 = “Always use”) was provided as answer options. Participants were coded as consistently using condoms if they reported always use a condom during sex.

#### Alcohol consumption and cigarette smoking

Participants were coded as (a) frequent drinkers if they reported having had 10 or more episodes of drinking in the past month [52]; (b) frequently intoxicated if they reported got drunk three or more times in the past month [53]; (c) binge drinkers if they had an equivalent of 5 drinks at one occasion in the past month; (d) problem drinkers if they experienced any of the following drinking-related issues: fighting with others, trouble with work, significant mistakes in performing a task, or accident and injury after drinking.

With regard to tobacco use, participants were coded as daily smokers if they reported smoking on 30 days in response to the question: “Please think back the past 30 days. During this period including today, on how many of the days (number of days) did you smoke cigarettes?”

### 2.6 Sample weights and statistics

Sample weights were assessed at the geounit, household, and individual levels. The household sampling rates were computed as the ratio of the households sampled over the total households within each geounit; the individual person’s sampling rates were computed as the ratio of persons sampled over the total eligible persons in each household. The challenge for this study is to estimate the geounit sampling rate, because the *residential area* of a district, although conceptually clear, cannot be practically determined with accuracy. We thus developed *the population-area substitution method*. Briefly, the ratios of the total persons (*P*_*g*_) over the area size (*Ag*) of the sampled geunits within a district was used as an estimate of the ratio of the total population (*P*_*d*_) and area size (*A*_*d*_) of the urban district. With *n* geounits selected from one district, the census data for *P*_*d*_, and the GIS data *A*_*d*_, the “true” residential area *A*_*r*_ of a district was estimated as the median of *P*_d_*A_*g*_/*P*_*g*_/*n*. With the estimated *A*_*r*_, the *sample weight* for the *i*th geounit in a district was computed as the ratio of *A*_*r*_ */A*_*g*_(i).

The survey estimation method for multi-stage random sampling design was used for statistical analysis to obtain accurate point estimate, standard errors, and 95% confidence intervals considering the stratification (districts) and clustering (geounits) unequal sample sampling probability and unequal sample weights [54, 55]. We used PROC SURVEYMEAN, the standard procedure for survey mean estimation from SAS 9.4 (SAS Institute, Cary, NC) to specify strata (district), cluster (geounit) and computed sample weights to obtain point estimate and 95% CI for the study variables. In addition to describing the measurement precision, the 95% CI was also used to compare group differences in the number of MSM and risk behaviors between migrant and non-migrant samples with no overlap in the 95% CI as evidence of significant differences at p<0.05 level.

## 3 Results

### 3.1 Characteristics of study sample

Results in Table 1 indicate that the total sample compromised 33.29% rural-to-urban migrants, 33.76% rural residents, and 32.95% urban residents. Relative to urban residents, rural migrants were younger and less educated; relative to rural residents, migrants were younger, better educated.

**Table 1:** Demographic Characteristic of the Three Random Subsamples.

Variables	Rural residents	Rural migrants	Urban residents	Total
**Total sample, n (%)**	580 (33.76)	572 (33.29)	566 (32.95)	1718 (100.00)
**Age (in years)**				
Range	18–45	18–45	18–45	18–45
Mean (SD)**	36.07 (7.91)	32.14 (8.16)	34.86 (7.62)	34.36 (8.06)
**Marital status, n (%)** **				
Married	488 (84.14)	411 (72.11)	410 (72.44)	1309 (76.28)
Unmarried	92 (15.86)	159 (27.89)	156 (27.56)	407 (23.72)
**Educational attainment, n (%)** **				
Primary or less	91 (15.69)	57 (10.00)	24 (4.24)	172 (10.02)
Middle school	349 (60.17)	304 (53.33)	122 (21.55)	775 (45.16)
High school	122 (21.03)	169 (29.65)	204 (36.04)	495 (28.85)
College or more	18 (3.10)	40 (7.02)	216 (38.16)	274 (15.97)
**Income (Yuan** ^a^ **), n (%)** **				
<1000	150 (25.86)	61 (10.66)	101 (17.84)	312 (18.16)
1000–2000	176 (30.34)	220 (38.46)	236 (41.70)	632 (36.79)
2000–4000	181 (31.21)	230 (40.21)	157 (27.74)	568 (33.06)
>4000	73 (12.59)	61 (10.66)	72 (12.72)	206 (11.99)

^a^; The income was measured on a monthly basis for rural migrants and urban residents and an annual basis divided by 10 for rural residents; 6 Chinese Yuan ≈ $1.

*: p < .05 and

**: p < .01

### 3.2. Comparison of rural migrants with non-migrant rural and urban residents

Results in the upper part of Table 2 indicate that most migrants migrated to 2–3 cities and had an average of 12.0 (95% CI [11.1, 12.8]) years of migration experience. Approximately 8% of the migrants had not visited home during the past year, and 86.9% (95% CI [82.2, 91.6]) had sent money home. Relative to urban residents, rural migrants were more likely to move, to live alone in new town or suburban area, and to perceive fewer urban residents in their neighborhood. Relative to non-migrant rural residents, rural migrants were also more likely to move, to live alone, to report a safe neighborhood, and to describe easy access entertainment installments.

**Table 2:** Migration Experience and Comparison of Living Condition, Neighborhood Environment and HIV-related Risk Behaviors among Rural Migrants and Non-migrant Urban and Rural Residents, Mean or % [95% CI].

Variables	Rural migrants	Urban residents	Rural residents
**Migration experiences**			
*No*. *of cities migrated*, *%*			
1 city	25.9 [20.9,30.8]	N/A	N/A
2–3 cities	43.6 [38.0,49.2]	N/A	N/A
≥4 cities	30.5 [25.1,36.0]	N/A	N/A
*Years migrated to Wuhan*			
Median [IQR]	10.0 [3.5, 15.7]	N/A	N/A
*No*. *of home visits in the past year*, %			
0 time	8.1 [5.5,10.8]	N/A	N/A
1–2 times	34.0 [28.1,39.9]	N/A	N/A
≥3 times	57.9 [52.1,63.7]	N/A	N/A
*Sending money home*			
Yes	86.9 [82.2,91.6]	N/A	N/A
*Intention to move*, %			
Likely	31.9 [27.7,36.2]	9.2 [6.8,11.6]	14.2[10.4,17.9]
Unsure	13.5 [10.6,16.5]	10.1[7.3,12.8]	12.9[9.0,16.9]
Unlikely	54.5 [49.8,59.3]	80.8[77.3,84.3]	72.9[67.8,78.0]
**Living conditions**			
*Residential locations*, %			
Old town	41.4 [36.2,46.7]	55.8 [51.6,60.0]	N/A
New town	39.0 [34.3,43.8]	18.3 [15.0,21.6]	N/A
Rural-urban joint zone	9.7 [7.0,12.5]	21.0 [17.6,24.5]	N/A
Suburban	9.8 [7.1,12.5]	4.9 [3.7,6.1]	N/A
*Housing ownership*, %			
Owner	17.2 [14.1,20.3]	70.1 [66.3,73.8]	N/A
Rental	67.6 [62.9,72.3]	11.6 [9.0,14.3]	N/A
Others	15.2 [11.1,19.3]	18.3 [15.2,21.5]	N/A
*Living arrangement*, %			
Living alone	50.0 [44.9,55.2]	40.5 [36.2,44.8]	33.7[28.2,39.1]
With others	50.0 [44.8,55.1]	59.5 [55.2,63.8]	66.3[60.9,71.8]
**Neighborhood environment**			
*Perceived safety*, %			
Safe	68.9 [64.1,73.7]	63.5 [59.5,67.6]	55.4[49.5,61.2]
Unsure	24.4 [19.8,29.0]	30.0 [26.2,33.9]	33.6[28.3,38.9]
Unsafe	6.7 [4.7, 8.6]	6.4 [4.2, 8.7]	11.0[7.6,14.4]
*Perceived urban residents around*, %			
Less than half	30.6 [26.8,34.5]	13.1 [10.0,16.2]	N/A
A half	16.4 [11.4,21.4]	15.9 [12.4,19.3]	N/A
More than a half	53.0 [47.6,58.4]	71.0 [67.0,75.1]	N/A
*Presence of prior acquaintances*, %			
Yes	73.0 [68.2,77.9]	74.2 [70.3,78.2]	N/A
*Existence of entertainment venues*, %			
None	12.5 [9.2,15.7]	12.2 [9.3,15.1]	69.2[64.4,74.0]
Some	60.5 [55.4,65.5]	58.9 [54.5,63.2]	26.7[22.3,31.1]
A lot	27.1 [22.3,31.8]	28.9 [25.1,32.8]	4.1 [1.5, 6.6]
*Accessibility of entertainment venues*, %			
Convenient	58.5 [53.7,63.3]	60.1 [56.0,64.2]	24.7[19.8,29.7]
Unsure	27.1 [23.2,30.9]	24.6 [21.2,28.0]	23.2[18.2,28.3]
Inconvenient	14.4 [11.4,17.4]	15.3 [12.2,18.4]	52.1[46.2,57.9]
**HIV-related risk behaviors**			
*Engage in commercial sex*			
Yes	5.6 [4.7, 6.5]	4.9 [3.1, 6.7]	3.0 [1.2, 4.7]
*Consistent condom use*			
Yes	24.1 [20.1, 28.1]	23.7 [18.9, 28.5]	13.4 [6.9, 19.9]
*Frequent drinkers*			
Yes	24.7 [21.3, 28.0]	25.4 [22.0, 28.9]	36.9 [33.1, 40.7]
*Frequent alcohol intoxicated*			
Yes	45.1 [41.3, 48.9]	48.8 [45.0, 52.5]	55.9 [51.9, 59.8]
*Binge drinkers*			
Yes	19.1 [16.0, 22.2]	28.4 [25.0, 31.9]	24.7 [21.2, 28.1]
*Problem drinkers*			
Yes	54.1 [49.2, 59.1]	46.3 [41.9, 50.7]	49.1 [43.3, 54.9]
*Daily smokers*			
Yes	32.6 [28.1, 37.2]	42.2 [37.9, 46.5]	42.1 [36.1, 48.0]

**Note**: Estimated with data collected from random samples. An exclusive 95% CI of a measure indicates a significant difference at p < .05 level between the rural migrant sample and the non-migrant urban and rural resident samples. N/A: Not applicable because the data were not collected for non-migrants.

Results in the lower part of Table 2 indicate that rural migrants did not differ from urban residents in most sexual risk behaviors and alcohol use measures. But rural migrants were less likely than urban residents to engage in binge drinking and to smoke daily. Relative to rural residents, rural migrants were more likely to use condoms consistently and less likely to drink and less frequently to get intoxicated.

### 3.3 The number of MSM and their characteristics

Among the rural migrant sample (Table 3), 5.8% (95% CI [4.7, 6.8]) were MSM, significantly more than those among the urban (2.8%, 95% CI [1.2, 4.5]) and rural (1.0%, 95% CI [0.0, 2.4]) residents. Migrant MSM were older, more likely to be married, better educated, migrated to more cities with more years as a migrant, and more frequent home visits. Migrant MSM were also more likely to co-habit with others in rental properties in new town, and perceived more urban residents but fewer prior acquaintances and more entertainment venues in the neighborhood.

**Table 3:** The Proportion of MSM among Rural-to-Urban Migrants Compared to that among Non-Migrant Urban and Rural Residents, Overall and by Demographic and other Characters, % [95% CI].

Variables	Rural migrants	Urban residents	Rural residents
**Total**	5.8 [4.7,6.8]	1.0 [0.0,2.4]	2.8 [1.2,4.5]
**Age**			
18–35	0.7 [0.0,1.5]	0.2 [0.0,0.5]	3.3 [0.4,6.1]
36–45	12.9 [10.4,15.3]	1.6 [0.0,3.9]	2.7 [0.8,4.6]
**Marital status**			
Married	6.9 [5.6,8.1]	1.2 [0.0,3.0]	2.7 [1.0,4.4]
Unmarried	1.8 [0.0,3.9]	0.2 [0.0,0.5]	4.1 [0.0,8.8]
**Educational attainment**			
Middle school or less	1.6 [0.1,3.2]	3.2 [0.0,7.9]	3.7 [1.6,5.9]
High school or more	10.8 [9.2,12.4]	0.1 [0.0,0.3]	0.0 [0.0,0.0]
**Monthly income**			
≤ 2000 Yuan	12.8 [9.9,15.6]	1.2 [0.0,3.5]	3.3 [1.2,5.3]
> 2000 Yuan	0.5 [0.0,1.2]	0.6 [0.0,1.8]	2.4 [0.0,5.0]
**No. of cities migrated**			
<2 cities	2.2 [0.0,4.5]	N/A	N/A
2+ cities	7.0 [5.6,8.4]	N/A	N/A
**Years migrated to Wuhan**			
0–10 years	1.1 [0.0,2.1]	N/A	N/A
>10 years	9.1 [7.2,11.1]	N/A	N/A
**No. of home visits in the past year**			
≤ 2	2.0 [0.0,4.1]	N/A	N/A
> 2	8.5 [7.2,9.8]	N/A	N/A
**Residential locations**			
Old town	1.4 [0.0,3.2]	1.6 [0.0,4.1]	N/A
New town	11.9 [10.2,13.6]	0.5 [0.0,1.1]	N/A
Rural-urban joint zone	2.1 [0.0,5.4]	0.0 [0.0,0.0]	N/A
Suburban	3.2 [0.0,7.6]	0.0 [0.0,0.0]	N/A
**Housing ownership**			
Rental	7.1 [6.2,8.0]	2.4 [0.0,6.4]	N/A
Non-rental	2.9 [0.2,5.6]	0.8 [0.0,2.3]	N/A
**Living arrangement**			
Living alone	1.9 [0.1,3.7]	0.8 [0.0,2.0]	2.4 [0.6,4.1]
With others	9.6 [8.2,11.1]	1.1 [0.0,3.3]	3.1 [0.8,5.3]
**Perceived urban residents around**			
Up to a half	1.5 [0.3, 2.7]	2.4 [0.0,6.9]	N/A
More than a half	9.5 [7.6,11.3]	0.4 [0.0,1.1]	N/A
**Presence of prior acquaintances**			
No	16.9 [13.3,20.5]	2.6 [0.0,7.6]	N/A
Yes	1.6 [0.4,2.9]	0.4 [0.0,1.1]	N/A
**Existence of entertainment venues**			
No	0.1 [0.0,0.3]	0.4 [0.0,1.2]	2.3 [0.4,4.3]
Yes	6.6 [5.3,7.8]	1.1 [0.0,2.7]	4.0 [1.1,6.9]

**Note**: Estimated with data collected from random samples. An exclusive 95% CI of a measure indicates a significant difference at p < .05 level between the rural migrant sample and the non-migrant urban and rural resident samples. N/A: Not applicable because the data were not collected for non-migrants.

### 3.4 HIV-related risk behaviors among MSM

Results in Table 4 indicate that among the rural migrant sample, the MSM migrants were significantly more likely than the non-MSM migrants to engage in commercial sex (79.2% vs. 1.1%) and less likely to have used a condom (9.3% vs. 28.2%). MSM migrants were also significantly more likely to engage in problem drinking and cigarette smoking. Similar risk patterns were observed for non-migrant urban and rural residents with regard to commercial sex and condom use, but the differences were smaller in scale.

**Table 4:** Differences in HIV-related risk behaviors between MSM and Non-MSM among rural migrants and non-migrant urban and rural residents, % [95% CI].

Variables	Rural migrants (n = 572)		Urban residents (n = 566)		Rural residents (n = 580)	
	MSM	Non-MSM	MSM	Non-MSM	MSM	Non-MSM
**Commercial sex**						
Yes	79.2 [66.6,91.8]	1.1 [0.4, 1.8]	3.5 [0.0,11.8]	4.9 [3.1,6.7]	5.2 [0.0, 13.2]	2.9 [1.1, 4.7]
**Consistent condom use**						
Yes	9.3 [5.7, 12.9]	28.2 [22.4, 34.0]	0.0 [0.0,0.0]	24.9 [19.8, 30.1]	3.4 [0.0,10.0]	14.9 [7.3, 22.6]
**Frequent drinkers**						
Yes	26.7 [6.7,46.6]	24.6 [21.2,28.0]	50.0 [0.9,99.1]	25.3 [21.8,28.7]	45.8 [26.4,65.3]	36.5 [32.6,40.4]
**Frequent alcohol intoxicated**						
Yes	53.3 [29.8,76.8]	44.9 [41.0,48.7]	50.0 [0.9,99.1]	48.8 [45.0,52.5]	66.7 [48.0,85.3]	55.4 [51.4,59.4]
**Binge drinkers**						
Yes	20.0 [0.1,39.9]	19.0 [15.9,22.2]	0.0 [0.0,0.0]	28.6 [25.2,32.1]	45.8 [26.4,65.3]	23.7 [20.3,27.2]
**Problem drinkers**						
Yes	86.2 [74.3,98.2]	52.2 [46.9,57.5]	8.3 [0.0,24.2]	46.7 [42.3,51.1]	45.0 [17.6, 72.3]	49.2 [43.3,55.1]
**Daily smokers**						
Yes	80.3 [67.8,92.9]	29.7 [24.9,34.5]	27.8 [0.0,78.4]	42.4 [38.0,46.7]	35.7 [5.3, 66.1]	42.3 [36.2,48.3]

**Note**: Estimated with data collected from random samples. An exclusive 95% CI of a measure indicates a significant difference at p < .05 level between the rural migrant sample and the non-migrant urban and rural resident samples

## 4 Discussion and Conclusions

To the best of our knowledge, this is the first study employing a random sample strategy to investigate MSM among rural migrants in China, including an estimation of the number of MSM, their risk behaviors and influential factors. Findings of this study add new data to advance the understanding of rural-to-urban migrants, migrant MSM, as well as their role in the spread of HIV through risk behaviors. Such findings are important for public health planning and decision making and for the development of intervention programs targeting rural migrants and migrant MSM for HIV prevention and control in Wuhan, as well as other cities in China with similar socioeconomic conditions.

### Significantly more MSM among rural migrants with high prevalence of HIV risk behavior

Results of this study show that it is *not* the HIV risk behaviors practiced by the rural-to-urban migrant population in general but the high proportion of MSM that renders them an at-risk population for HIV infection. The proportion of MSM among migrants was twice as high as that among rural residents and approximately 6 times that among urban residents in Wuhan. Furthermore, HIV risk behaviors were also more prevalent among MSM migrants than among non-MSM migrants, consistent with other study findings [34]. Based on our estimates in this study and the population statistics[23, 48, 56, 57], the estimated number of MSM migrants in Wuhan and China would be 58 000 (95% CI [47 000, 68 000]) and 10 million (95% CI[8,12]) respectively. Assuming the same recorded prevalence rate of 6.3% HIV infection among the MSM in general [9], an estimate of 3650 (95% CI [2960, 4284]) migrant MSM in Wuhan, and 655 000 (95% CI [529 000,768 000]) in China who are living with HIV. The reported total persons living with HIV (including those who are MSM) is 4967 in Wuhan [58] and 437 000 in China [59].

### Characteristics of MSM among rural-to-urban migrants

Findings of this study reveal that demographically, migrant MSM, relative to non-MSM tended to be married, older, and better educated, inconsistent with other studies in which the MSM were younger and unmarried [60]. The inconsistency could be due to the fact that the other studies are all based on convenience samples, in which younger, non-married MSM actively engaging in sex may be more likely to be recruited [61].

The married migrant MSM warrant particular attention. Many MSM in China marry a woman following the traditional cultural norms [25, 62, 63]. Married migrant MSM represent a great risk for cross gender and cross rural-urban HIV infection. Consistent with reported studies, migrant MSM relative to non-MSM are more likely to move to multiple cities, and make more frequent home visits [60], which facilitate cross-boundary and large-scale HIV transmission.

Another important finding of this study is that migrant MSM were more likely to co-habit with others in rental property located in new rather than established towns. They are also more likely to report living in urban residencies but less likely to report knowing any of their neighbors. This finding suggests the significance of social capital, informal monitory and collective efficacy [64, 65] in regulating health risk behaviors among rural MSM for HIV prevention.

### Implications for HIV/AIDS control in Wuhan and similar cities in China

Findings of this study indicate that rural-to-urban migrants in Wuhan remain a high-risk population. It consists of the highest proportion of MSM with increased likelihood to engage in an array of behaviors related to HIV infection, including substance use/abuse and having sex with high risk partners. In addition to exposing themselves to increased risk of HIV infection, these migrants frequently move between rural and urban areas facilitating rural-urban transmission of the virus. Effective control of the HIV epidemic in Wuhan could be difficult without persistent and effective measures protecting this high risk population.

The findings from out study with data collected in Wuhan may also applicable to many other inner cities in China with similar socioeconomic conditions. Different from the coastal cities like Hong Kong, Shanghai, and Shen Zhen, Wuhan is located in central China with a medium level of economic development. Wuhan is also known as the transportation hub in China where people migrate from almost all directions through Wuhan.

Findings of this study suggest two intervention strategies. The first strategy is the continuation of the promotion of condom use among MSM. Sexual risk behaviors are prevalent among this population, and the rate of condom use was rather low [66]. MSM can be educated to take protective measures against HIV infection. Findings from a meta-analysis of 22 interventions research among MSM in China [67] indicate significant effect in enhancing HIV/AIDS knowledge with effect size d [95%CI] = .627[.460,.793] and condom use with d = .394[.274,.541]. Findings of this study provide data supporting more effective measures to educate migrants and migrant MSM regarding HIV/AIDS and to encourage condom use.

Another strategy is to promote social wellbeing through public policy and community organizations. Findings of our study indicate that separation from spouse and family and reductions in social connection/capital and living in neighborhoods with entertaining venues play a role in the increased prevalence of HIV risk behaviors. One intervention approach would be to organize family reunions for those migrants who are either married or engaged to get together on periodical basis. Such reunion can also be used as a venue to distribute HIV knowledge and condom skills.

Another approach would be social capital-based intervention as has been used in African countries to enhance social capital and community cohesion through organized and small group-based activities [68, 69]. Study findings in China indicated a positive relationship between social capital and mental health [32, 33, 64, 70]. To prevent the HIV epidemic mediated through migrants, particularly migrant MSM, group-based activities can also be organized to enhance the interaction within migrants and between migrants and urban residents. Such social capital investment activities should be able to help migrants to form adequate social capital in urban settings for better informational, emotional, and instrumental support, reducing the likelihood to engage in HIV risk behaviors.

The limitations to this study include: (a) data for analysis were collected in one city in China. Caution is needed in generating the findings of this study to other cities within China; (b) the total number of MSM is relatively small due to a population-based sampling, preventing this study from more in-depth analysis of various HIV risk behaviors; (c) rural resident sample did not cover all rural origins of the migrants in Wuhan; (d) data for the study are cross-sectional in nature, and therefore causal conclusion is not warranted.

Despite these limitations, this study is the first to investigate MSM among rural-to-urban migrants in China with a random sample. Findings of this study provide new data much needed to contain the HIV epidemic in China.

## Supporting Information

S1 Migrant MSM Dataset (CSV)
